# Supplementary material for: Description, Distribution, and Relevance of Viruses of the Forest Pathogen Gremmeniella abietina
Source: Viruses. 2018 Nov 20;10(11):654. doi: 10.3390/v10110654 (PMC6267220; doi:10.3390/v10110654)
Supplement: Supplementary file 1 [file viruses-10-00654-s001.pdf]

Supplementary data

Materials and Methods

dsRNA extraction and sequence determination

The Spanish isolates P3-7 and 06P of *Gremmeniella abietina* were chosen for the determination of possible viruses based on their dsRNA-banding pattern with 5 and 6 kb-bands not described previously. Approximately 3 g of freshly growing mycelium was collected and frozen with liquid nitrogen in 50 ml RNase-free tubes. Once all of the nitrogen was evaporated, the mycelium was homogenised with 8 mm-diameter stainless steel beads (Retsch, Germany). The tube was vortexed on a standard vortex mixer at maximum velocity for 3 min. Then, 2.5 ml of lysis buffer (2.5 ml TriseHCl pH 8.0 (Carl Roth GmbH þ Co. KG, Germany), 0.5 ml of 1 % b-mercaptoethanol (Serva Electrophoresis, Germany), 2.5 ml of EDTA (Penta, Petr Svec, Czech Republic), 15 ml of SDS (pH 7,2; Carl Roth GmbH þ Co. KG, Germany), and 25 ml of sterile distilled water (SDW) were added. The dsRNA-binding cellulose added was Sigmacell cellulose (C6288) (SigmaeAldrich; Germany). The protocols for double-stranded (ds) cDNA synthesis and cloning were developed as described in related works [24, 25, 26, 27] Sequencing of the cloned cDNA fragments and RT-PCR products for population analyses was conducted by Macrogen Europe. The universal primers M13 Forward (17 mer) and M13 Reverse (17 mer) were used. A set of specific primers to fill some gaps within the sequences as well as a T4 RNA- primer for sequencing the ends (Table S1). All of the obtained sequences were adjusted, compiled, and aligned using the Geneious Pro 5.5.9 software package (Bio- matters Ltd). NCBI Protein Blast (Blastx) was employed to search

for similar sequences and conserved domains. NCBI ORF Finder (http://www.ncbi.nlm.nih.gov/gorf/gorf.html) and Geneious Pro 5.5.6 were used to search for ORFs.

Table S1. Primers used in this study for the determination of the virus sequences

**Primer name**	**Primer sequence 5´- 3´**	**Primer type**
FU1_FW1	GCACTCAGTAGAGTTGCTTC	specific
FU1_RE1	ACGCTTCAGTCTATAACT	specific
T4	AAC CCG GGT CGA ATG C	Universal
M13F	CAC GAC GTT GTA AAA CGA C	Universal
M13R	GGA TAA CAA TTT CAC ACA GG	Universal
